# The challenges of recognising individuals with few distinguishing features: Identifying red foxes *Vulpes vulpes* from camera-trap photos

**DOI:** 10.1371/journal.pone.0216531

**Published:** 2019-05-09

**Authors:** Jo Dorning, Stephen Harris

**Affiliations:** School of Biological Sciences, University of Bristol, Bristol, United Kingdom; University of Agricultural Sciences and Veterinary Medicine Cluj-Napoca, Life Science Institute, ROMANIA

## Abstract

Over the last two decades, camera traps have revolutionised the ability of biologists to undertake faunal surveys and estimate population densities, although identifying individuals of species with subtle markings remains challenging. We conducted a two-year camera-trapping study as part of a long-term study of urban foxes: our objectives were to determine whether red foxes could be identified individually from camera-trap photos, and highlight camera-trapping protocols and techniques to facilitate photo identification of species with few or subtle natural markings. We collected circa 800,000 camera-trap photos over 4945 camera days in suburban gardens in the city of Bristol, UK: 152,134 (19%) included foxes, of which 13,888 (9%) contained more than one fox. These provided 174,063 timestamped capture records of individual foxes; 170,923 were of foxes ≥ 3 months old. Younger foxes were excluded because they have few distinguishing features. We identified the individual (192 different foxes: 110 males, 49 females, 33 of unknown sex) in 168,417 (99%) of these capture records; the remainder could not be identified due to poor image quality or because key identifying feature(s) were not visible. We show that carefully designed survey techniques facilitate individual identification of subtly-marked species. Accuracy is enhanced by camera-trapping techniques that yield large numbers of high resolution, colour images from multiple angles taken under varying environmental conditions. While identifying foxes manually was labour-intensive, currently available automated identification systems are unlikely to achieve the same levels of accuracy, especially since different features were used to identify each fox, the features were often inconspicuous, and their appearance varied with environmental conditions. We discuss how studies based on low numbers of photos, or which fail to identify the individual in a significant proportion of photos, risk losing important biological information, and may come to erroneous conclusions.

## Introduction

Biologists have long used hand-held cameras for photographic identification (photo ID) of animals [1]; individuals have been identified from unique dorsal fin shapes [2–4] or pelage patterns [5–8]. Photo ID is often easier using hand-held cameras because they can be adjusted to improve image quality, and repeated recaptures of the same individual from different perspectives produces an image archive with which to compare new sightings.

Over the last twenty years, camera traps have revolutionised the ability of biologists to study species that are rare, elusive, and live in remote areas, and provide a cost-efficient method for faunal surveys [9]. They are also widely used to estimate population densities [10], although many studies fail to acknowledge or report study design details that affect population estimates [11–15], or recognise that the emission of sound and light by camera traps can influence the behaviour of the animals being studied [16,17].

While camera traps can also be used for individual identification, photo quality can be poor compared to hand-held cameras, since motion-triggered cameras are less easily adjusted and often produce blurred, poorly-exposed or partial images. This can be problematic for studies that require accurate identification of individuals [18–20]. Consequently, photo ID from camera-traps is generally used for species with easily distinguished natural markings such as spot or stripe patterns, or antler configurations [21–26], which are less reliant on image quality.

Processing the large number of photos generated in camera-trap studies can also be challenging. Inexperienced observers can be used to identify species [27] and individuals of species with obvious natural markings [28], as can image-recognition software [5,29–32]. Automatic software can also be used to identify, count and describe the basic behaviours of some species [33] and software developed for human face detection and identification has been used to identify individual chimpanzees *Pan troglodytes* [34].

However, identifying individuals of subtly-marked species remains challenging: distinctive features are limited in number, less easily discernible in photos of low quality or from suboptimal angles, and may change in appearance over time, all of which increase the risk of misidentification [35–37]. Larger samples of more animals may preclude photo ID by further reducing the number of unique markings [10,29] and/or require additional manpower to process, although increasing the number of observers introduces inconsistency [38]. False matches, when two photos of different individuals are incorrectly identified as being the same animal, or false mismatches, when two photos of the same individual are wrongly identified as different animals, can deflate or inflate estimates of population density [35]. Several capture-recapture models account for false mismatches [35,39–41], but false matches are considered rarer and less easily controlled for [10]. While error rates in identification can be accounted for in population models, they have a significant effect on estimates of survival [42] and lead to false inferences when investigating individual differences in ecology and behaviours such as scent marking [19] and dispersal [43].

Consequently, low confidence in photo IDs of subtly-marked species has often precluded their study with camera traps [44–46]. While photo ID could be assisted by trapping and marking individuals using tags, collars or shaved patches of fur [38,47,48], live-trapping a significant proportion of a study population may not be feasible and negates the perceived non-invasive benefits of camera trapping, but see [16,17]. Therefore, many researchers use targeted camera positioning, sometimes with lures (food and/or scent), to enhance photo ID, e.g. placing cameras along trails to capture coyotes *Canis latrans* in flight posture [49]; photographing striped skunks *Mephitis mephitis* from above rather than laterally [50]; using paired camera stations for bilateral identification of cougars *Puma concolor* [51]; placing scent lures to obtain multiple photos of red foxes *Vulpes vulpes* from different positions [52]; and elevating lures to encourage wolverines *Gulo gulo* [53] and American martens *Martes americana* [54] to expose their unique ventral coat patterns. However, lures can alter ranging patterns [55], increase disease transmission [56], and bias population density estimates if individuals are differentially attracted or repelled [57,58].

The red fox is the most widespread terrestrial mammal [59] and of global importance as an invasive species, predator, competitor and vector of disease. Foxes colonized British cities in the late 1930s and live at higher densities than their rural counterparts [60,61]. Since urban foxes have habituated to anthropogenic activity, they are less wary of people and so cities provide an ideal opportunity to use camera traps to advance our knowledge of red fox ecology and behaviour. However, an earlier study claimed that photo ID was not reliable for red foxes and cautioned against the use of this technique for any species with few natural markings [62].

As part of a larger study of urban foxes, we conducted a two-year camera-trapping study to investigate individual variation in fox behaviour. A key requirement of this work was accurate photo ID. In this paper, our objectives are to (1) demonstrate how red foxes can be identified individually from camera-trap photos, (2) highlight camera-trapping protocols and identification techniques that facilitate photo ID of species with limited or subtle natural markings, and (3) examine the impacts of having too few photos and/or failing to identify the individual in a significant proportion of photos.

## Materials and methods

### Study area

We conducted our study in approximately 1.5 km^2^ in the northwest suburbs of Bristol, UK. The habitat consists predominantly of 1930s semi-detached housing with medium-sized/large gardens and has one of the highest densities of foxes in the city [63]. It is the site of an intensive study covering four decades and there is a long-term record of patterns of dispersal and reproduction, and changes in population density and social group structure based on radio-tracking and CMR data, e.g. [64–68].

### Camera trapping

**Fig 1:**
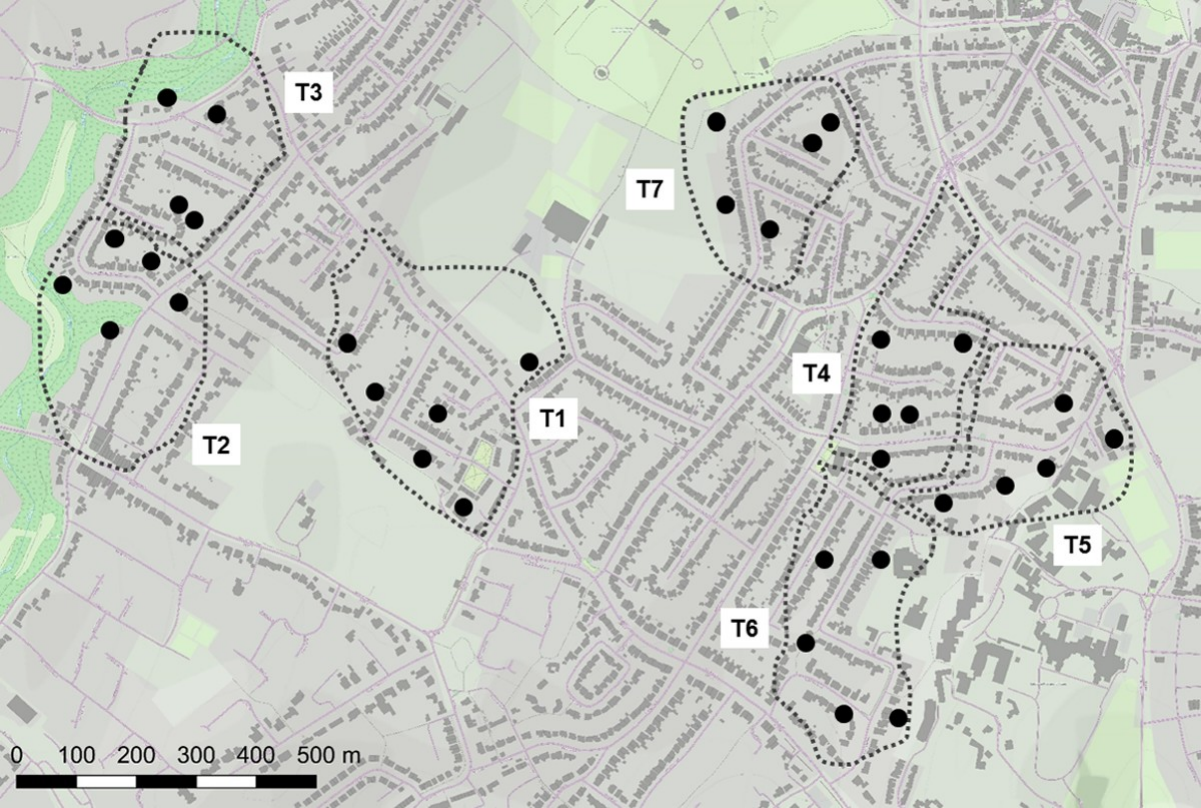
Camera-trap locations in seven fox territories (T1-T7) in Bristol. The estimated territorial boundaries are shown by dotted lines and camera-trap locations by black circles. The lighter grey indicates built up areas; darker grey buildings; and green open spaces such as parks, playing fields, cemeteries and allotments. The centre of the study area is 51.48623°N, 2.62468°W. Map drawn in QGIS with the OpenLayers plugin [71].

Pilot studies at three sites undertaken continuously for 80 days from March to May 2013 showed that a 40-day camera-trapping period would capture all the individuals on a territory without violating the assumptions of population closure [69]. So between July 2013 and June 2015 we positioned one camera trap in each of 4 to 6 privately-owned residential back gardens in each of seven fox territories (Fig 1): all householders gave permission for the study to be undertaken. The cameras were continuously active for 40 days in each of four consecutive seasons: spring (March-May; birth and early cub-rearing), summer (June-August; late cub rearing, onset of juvenile independence), autumn (September-November; onset of dispersal period) and winter (December-February; peak dispersal and mating). Consecutive surveys in the same territory were separated by a minimum of 39 days, so each seasonal survey was considered independent, and a finite number of foxes could be captured in each survey. Not all territories were surveyed concurrently due to logistical constraints [70].

We estimated territory boundaries using long-term radio-tracking data and topographical features such as roads [70,72]. We used one ScoutGuard SG565F-8M camera (Boly Inc., Santa Clara, CA 95051, USA) per garden to maximize territory coverage [11]. This model was selected because of its wide field of detection (52°), 26 m detection range, 22 m flash range, long battery life (up to 6000 flash pictures on 8 AA lithium batteries), and the white flash allowed us to collect high resolution (8 MP) colour photos day and night: these were essential for individual identification [45,73]. The 80-day pilot studies in 2013 confirmed that the flash and/or camera noise did not influence the behaviour of the foxes: they showed no visible reaction to either, and did not change their behaviour i.e. they continued to eat the food in situ rather than carry it away, and did not reduce the number or length of their visits [70]. This is probably because urban foxes encounter novel objects, flashing lights from traffic, household and other security lights, a variety of mechanical and other noises, and other anthropogenic activity on a daily basis.

To aid individual identification, we endeavoured to maximize the number of photos taken of each animal when it visited a garden: we set cameras to record three consecutive photos in the daytime (this was not possible at night when the flash was used), camera sensitivity to high, and the delay between consecutive exposures to zero. To avoid influencing fox behaviour, we only used gardens where householders already routinely fed the foxes more than twice a week, usually with household scraps or dog meat, and had an established history of fox provisioning. Any householders that changed provisioning practices during the study were eliminated in subsequent seasons; thus in some territories the number of camera traps varied between seasons [70].

We tied each camera trap to a tree or stake 40 to 70 cm above the ground, 1.5 to 3 m from the established feeding area: an established history of provisioning by the householders encouraged foxes to visit their garden and linger in a particular area. This facilitated camera positioning to improve image quality and increased capture rates by maximising the chances of getting multiple images of each fox from different angles and both sides. Cameras were angled parallel to the ground to maximize flash range, which aided the identification of foxes photographed further from the camera. We fitted flash diffusers to avoid overexposure where foxes were fed close to the camera and checked cameras weekly to replace batteries and memory cards, and avoid data loss from malfunction or dislodgment. We used Camera Base version 1.6 [74] to manage data and identify individuals.

### Individual identification

**Fig 2:**
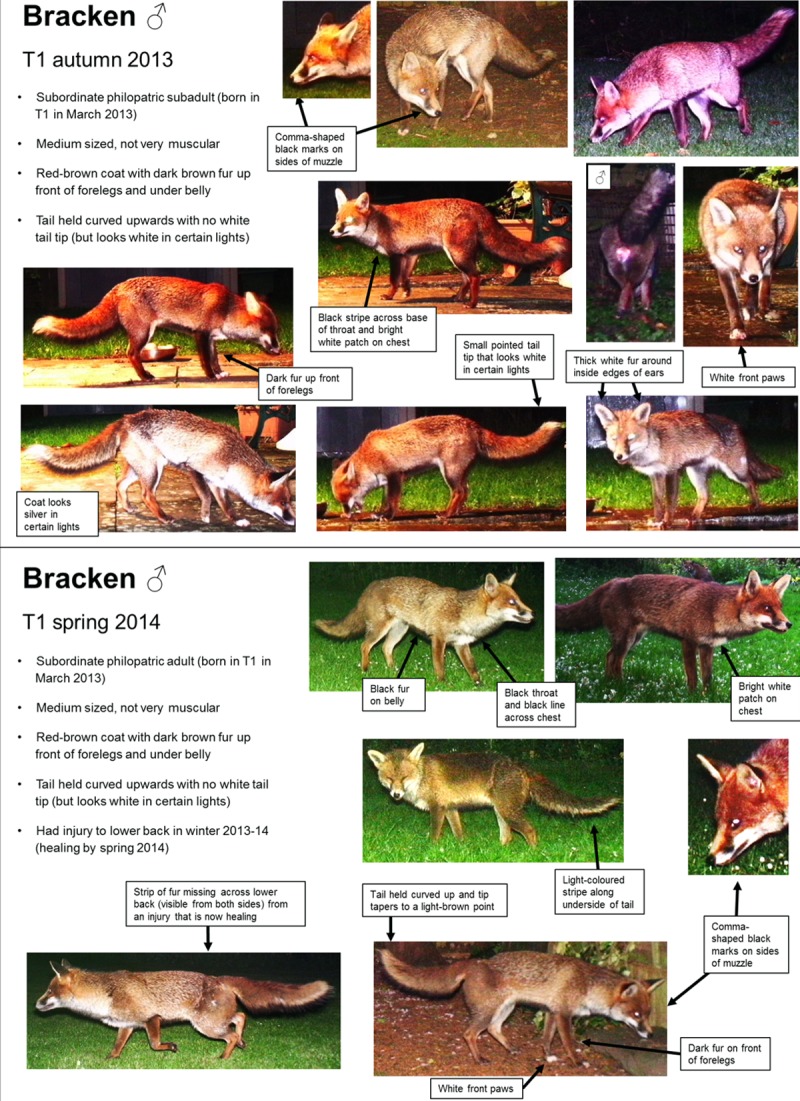
Example of autumn and spring identification sheets for one fox. These illustrate the multiple features used to ensure that it could be identified under different lighting conditions, from both sides, and when only some parts were visible on the camera-trap photo.

We used camera-trap photos collected during a two-week period in February 2013 and published photos [52] to select features that could be used to identify individual foxes (Table 1); the combination of features available and their location on the body varied between animals. Some key features, such as white fur on the feet, were only visible when the fox was standing on a hard surface or the lawn had been cut recently; compare, for instance, images of the same fox standing on different surfaces in Fig 2. Cubs were not identified individually in spring surveys as they still had their juvenile coats, which lack sufficient distinguishing features; younger animals are often more difficult to identify than adults [28,75].

**Table 1:** Morphological features selected for individual identification.

Feature	Sources of variation
Body size, build and condition	Posture; distribution of fat and muscle; leg length; depth of chest/belly; thickness of neck (older or more muscular foxes, particularly males, have thicker necks)
Body coat	Fur condition (thickness, coverage, shine); colour on body (ranges from dark brown-black to light silver-brown), belly (white, slate-grey, black) and chest (black or white patches or stripes)
Tail coat	Fur condition (thickness, bald patches, damage); colour and patterning (darker or lighter than body coat, striping, colour of tail tip); shape and size of dark patch around the supra-caudal gland
Tail shape	Length relative to the body; thickness; straightness; fur condition; tip shape (pointed, rounded, tapered, bulb-shaped, tufted, curled or flattened)
Head/face shape	Size of head; broadness of forehead; length of muzzle; fullness of cheeks
Muzzle	Scarring on top of nose; colour and shape of fur markings on each side of muzzle
Ears	Length; shape (rounded or pointed ear tips); colour and texture of fur inside ears; speckling on backs of ears; tears in ear edge
Leg markings	Height and shape of black socks on legs; black or white fur on fronts of thighs; fur colour on inner-sides of legs; fur speckling
Paw markings	Colour and patterning of fur, especially white marks
Injuries	Infection with sarcoptic mange; fresh/healing bite wounds; scars on the face and lower limbs; deformations, e.g. shortened or bent tails, crookedly healed fractures
Ear tags and collars where present	Tag colours; tag numbers if visible; tag position in each ear; collar colour; collar condition (curled strap ends, bent or missing aerial)

**Fig 3:**
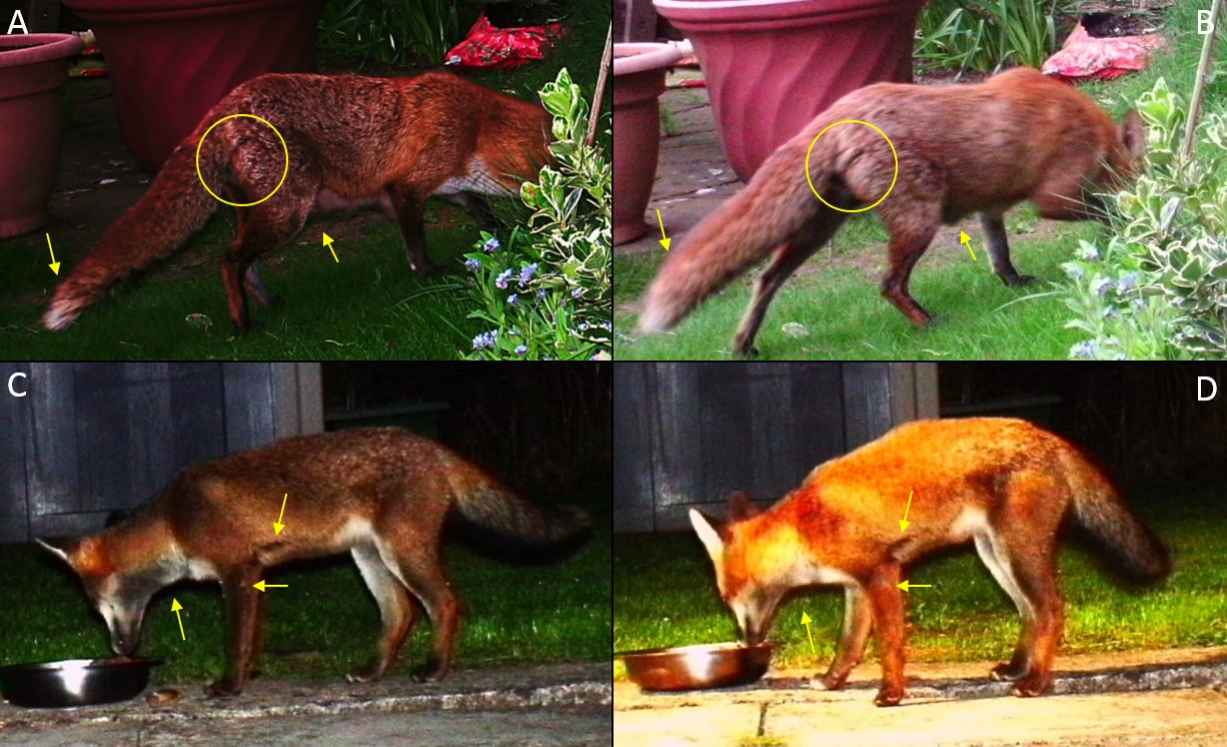
Illustration of how a fox’s appearance can vary with lighting conditions. Top row: photos of the same fox taken in the same location (A) with a flash at dusk and (B) without the flash in daylight. The coat colour appeared different but tail shape, signs of lactation and a dark line on the right hindquarter remained consistent. Bottom row: photos of another fox taken in the same location (C) with a flash in the dark and (D) with a flash when an outside light was also on in the garden. While the coat colour appeared different, the dark lines on the throat and above the elbow and a white spot on the left foreleg remained consistent.

As more photos were identified, we accumulated a library of images, which we used to compile individual identification sheets for each season to account for temporal changes in appearance (Fig 2). These sheets contained details of each individual’s distinguishing features and example photos taken from different angles and under different conditions, e.g. light and dark, wet and dry. We identified multiple distinguishing features for each fox, so that they could still be identified when certain parts of their body were not visible in a particular photo. Features became more obvious after viewing several photos of the same fox in different positions and under different light and environmental conditions (Fig 3), as it became easier to differentiate between permanent markings and those caused by shadows, reflections, dirt or moisture. Where possible, we determined sex from the genitalia and/or signs of lactation. This was easier in winter when males had swollen testes, and spring when females were lactating.

Morphological features were combined with spatiotemporal information from photo timestamps and camera-trap location to aid identification. We used the identification sheets to help match new photos with a known individual. First, we compared the photos with those of individuals captured in the same territory within the same day, week or 40-day survey and then, if no matches were found, with individuals captured at increasing spatial and temporal distance. Knowing the locations of territory boundaries allowed us to reduce the number of possible identities of each new image and we checked unexpected matches, such as foxes seen outside their usual territory, particularly carefully. Photo timestamps helped reduce the number of possible matches, as an individual could not be captured in two locations at the same time. We recorded each fox as either a new individual, a known animal, or unidentifiable. If a photo could not be identified, we recorded whether this was due to poor image quality or poor visibility of a key identifying feature, and whether this was due to body position, obstruction or a partial image.

All identifications were made by JD to ensure consistency [38]. Human visual identification improves with experience [28], so identifications were continuously reviewed throughout the photo ID process, particularly as more distinguishing features became apparent for each fox, and all photos were double-checked at least once after completing the photo IDs for each survey. We tested JD’s consistency by randomly selecting five surveys. JD then identified all the foxes photographed on the last day of each survey again without reference to her previous identifications.

### Testing the need for artificial marks

As part of a long-term study of Bristol’s foxes, some individuals had been marked with a different colour tag in each ear (Rototags, Dalton Tags, Newark, Nottinghamshire NG24 1BS); selected individuals were also marked with a VHF radio collar covered in coloured insulating tape. We used tag colour combinations and the presence of a collar and its colour as additional identifying features. To determine whether artificial marks aided photo ID, we randomly selected 10 foxes that were tagged but not collared; for each fox we counted the number of photos in one season where the tags were not visible and so could not be used to identify the individual. We then fitted a beta regression model with variable dispersion and a logit link function in R version 3.2.2 [76] using the package *betareg *[77] to test whether a greater proportion of photos could be identified in territories where a greater proportion of the foxes had been tagged. Diagnostic plots confirmed an acceptable model fit; we report χ² and *p*-values from a likelihood ratio comparison test between the full and null models.

### Impact of failure to identify the individual in a photo

To quantify the impacts of the number of photographs per individual and the failure to identify the individual in a proportion of photos on population estimates, we selected all animals ≥ 5 months old since these had reached dispersal age and were no longer tied to their natal range. We then randomly removed 10%, 20%, 30%, etc. to 90% of the photos taken in each season on each territory separately for residents and non-residents to simulate the effects of fewer photos per individual, and failing to identify the individual in a photo.

### Ethical statement

All feeding protocols were already in place in the gardens selected for the study. Householders supplied and replenished the food every 1–3 days at their discretion, as part of their normal fox-feeding routine.

The camera-trapping protocols were approved by the University of Bristol’s Animal Welfare & Ethical Review Board. No foxes were captured for this project; some had been ear-tagged and radio-collared as part of a long-term study. Most radio-collars were removed when they ceased to function: some foxes died before the collar was recovered and some collars dropped off (due to wear) prior to recovery. Animal capture and handling procedures followed the guidelines of the American Society of Mammalogists [78], were approved by the University of Bristol’s Animal Welfare & Ethical Review Board, and were licensed under the Animals (Scientific Procedures) Act 1986.

## Results

The 28 camera-trap surveys (7 territories, 4 seasons) totalled 4945 camera days: we collected circa 800,000 camera-trap photos. Of these, 152,134 (19%) included foxes, of which 13,888 (9%) contained more than one fox. These provided 174,063 timestamped ‘capture records’ of individual foxes, of which 170,923 were foxes ≥ 3 months old. JD was able to identify the individual in 168,417 (99%) of these capture records; the remainder could not be identified due to poor image quality (*N* = 492), or because key identifying feature(s) were not visible due to body position (*N* = 422), obstruction (*N* = 622) or a partial image (*N* = 970).

JD identified 192 different foxes ≥ 3 months old: 110 males, 49 females and 33 of unknown sex. Of these, 78 (41%) were identified in more than one season. A visit to a garden was defined as a sequence of photos of the same fox with a time interval of < 15 minutes between any two photos [70]; a time interval of > 15 minutes between two consecutive photos was taken to indicate the end and start of separate visits by the same fox [79]. Individuals were often photographed multiple times during each visit: median photos per visit = 3.1, range 1–17. We obtained between 1 and 8891 photos of each fox (mean = 877, median = 26) across the study period.

**Fig 4:**
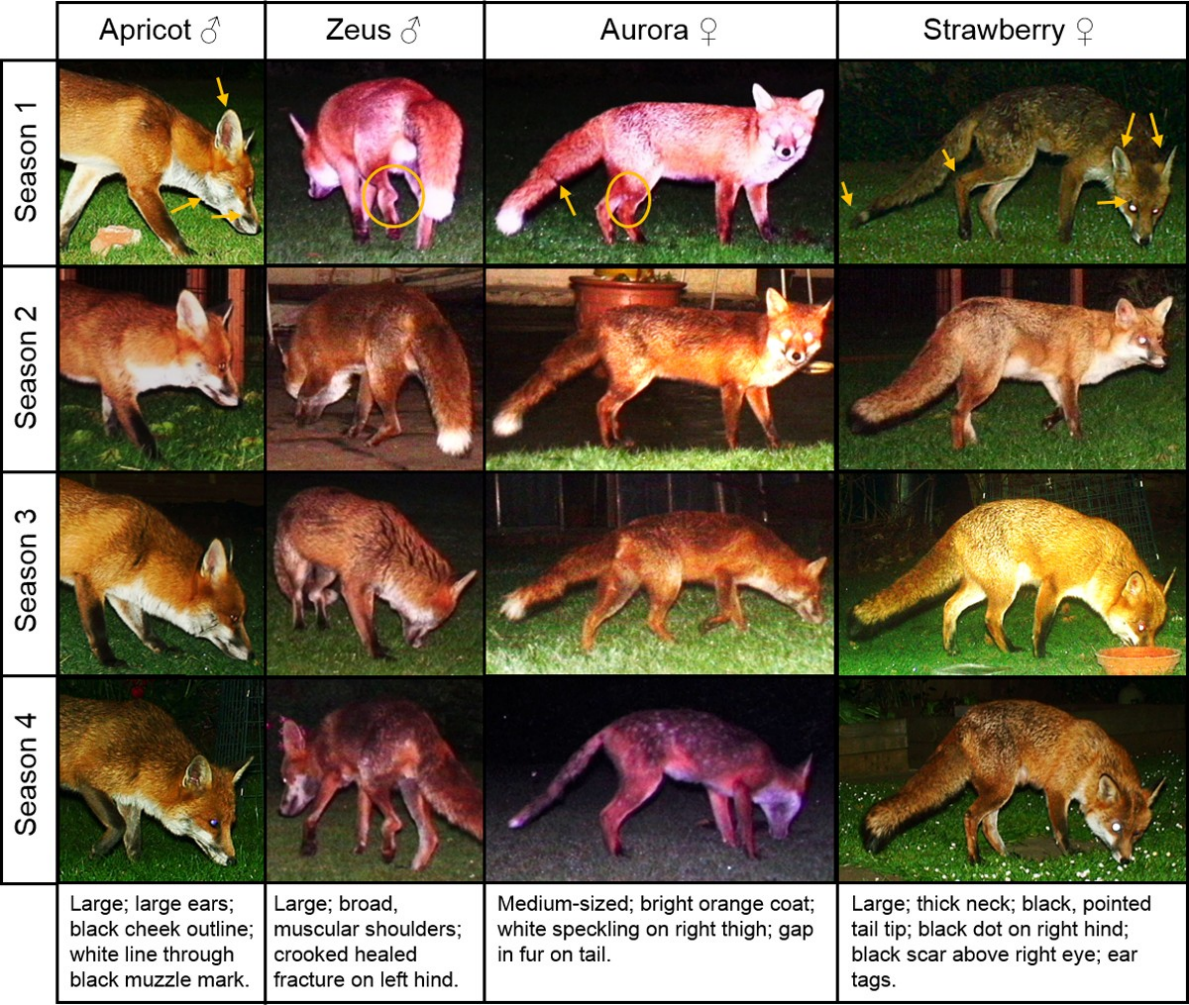
Temporal persistence of identifying features over consecutive seasons in four adult foxes. Seasons 1 to 4 are summer to spring for Apricot and Strawberry and autumn to summer for Aurora and Zeus. While coat thickness and fat deposition changed seasonally, other features were visible in every season.

The colour of the longer fur on the torso and tail showed more seasonal variation, and its appearance was more variable under different lighting conditions (Fig 3). The identifying features that persisted across seasons included body size, tail shape, permanent deformities such as healed fractures, and the colour of the short fur on the muzzle, chest and legs (Fig 4).

### Reliability of identification

Because these photo capture records were collected in parallel with a longer-term study, we had several measures to confirm JD’s accuracy and consistency of individual identification:-

1. Seven foxes were caught and tagged part-way through the study, so they were first identified without tags. Their identities did not change after being tagged i.e. they were not being confused with other individuals before they were tagged.
2. During the course of the study, 13 foxes contracted sarcoptic mange and 36 were injured (three of which also contracted sarcoptic mange). The injuries included bite wounds, broken limbs/obvious limps and damage to the tail. So 46/192 (24%) foxes acquired new injuries and/or sarcoptic mange during the study: the acquisition of new identification features confirmed that they had not been confused with other animals.
3. Four foxes (three of which were tagged) died during the study. The untagged fox was the dominant male on territory 6. He had received a serious injury, and we monitored the decline in his condition before he disappeared, presumably having died from his injuries. The deaths of these animals confirmed that they were not being confused with other group members, and the new individuals that invaded the territory to contest each vacancy were quickly recognised [70].
4. The number, and changes in numbers, of non-residents we identified on territories was consistent with known seasonal patterns of fox behaviour, and the origins of many of these animals could be established because they were recognised as residents on other territories [70,79].
5. Identifications were continuously reviewed, particularly as more distinguishing features became apparent, and all photos were double-checked at least once after completing the photo ID for each survey; very few IDs changed.
6. When JD blind-tested her identifications from the last day of five surveys, fox identity was the same in 714/722 photos (99% agreement).

Thus JD showed high levels of both accuracy and consistency in her identifications.

### Artificial marks

**Fig 5:**
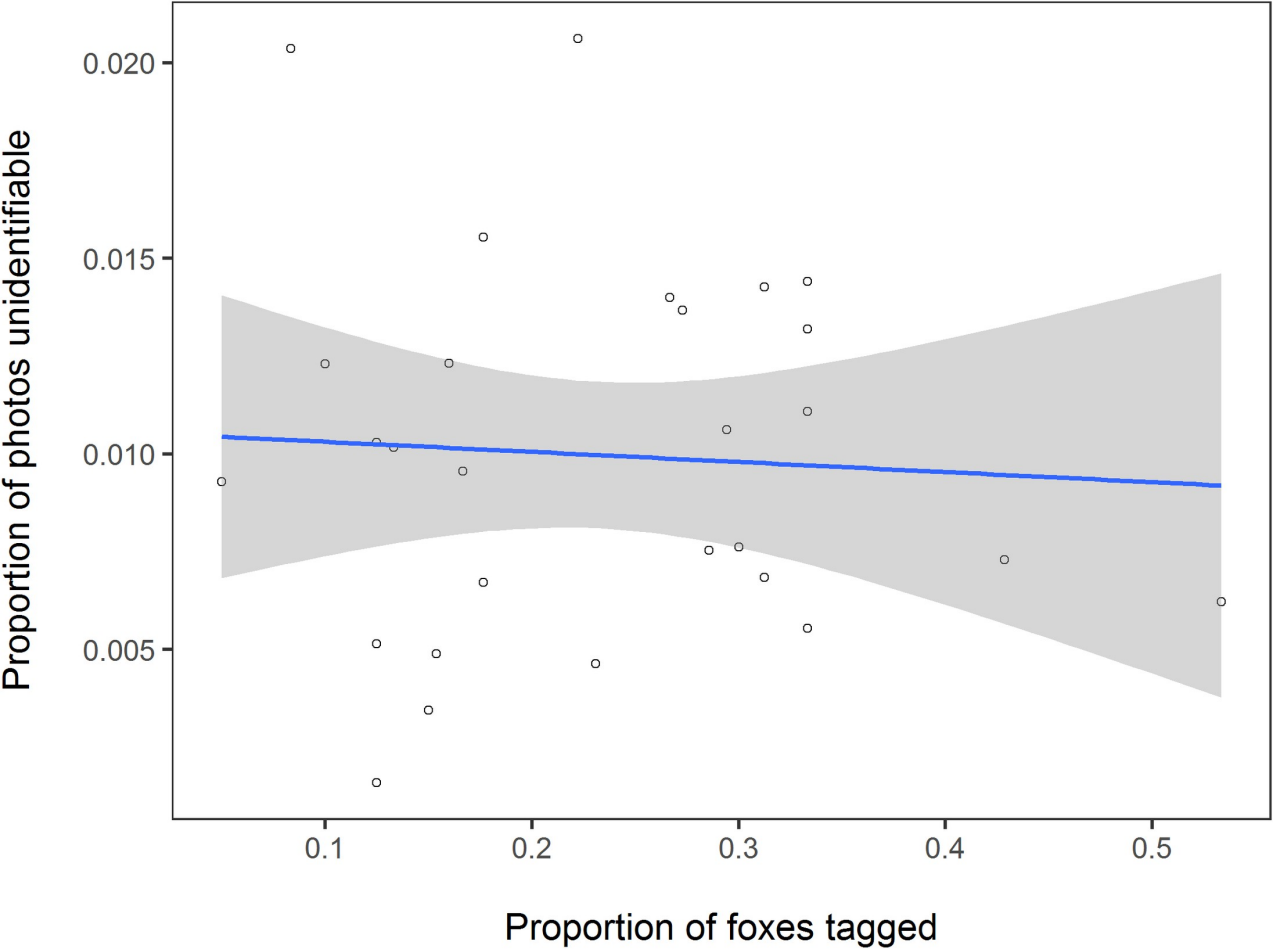
Relationship between the proportion of photos that were unidentifiable and the proportion of foxes ear-tagged on each territory. The circles denote each 40-day survey and the shaded ribbon the standard error.

Of the 192 identified individuals, 29 (15%) were marked with ear tags; four also had radio collars. We checked 5572 photos of ten tagged foxes from a random selection of surveys: the fox was identified from natural markings alone, i.e. when ear tags were not visible, in 20% of the photos. The proportion of photos where the tags were not visible ranged from 16 to 39% per individual (Table 2): there was no seasonal effect on the proportion of photos where the ear tags were not visible (one-way ANOVA, *F*_(3,6)_ = 1.294, *p* = 0.359). The proportion of foxes on a territory that were tagged had no effect on the proportion of photos where each fox could not be identified (χ²(2) = 4.738, *p* = 0.094; Fig 5). Thus tagging did not increase the likelihood that a fox was identified, but did make photo ID faster by adding to each individual’s combination of identifying features.

**Table 2:** The ten foxes with ear tags chosen from a random selection of surveys that were used to test the effects of artificial marks on the ability to identify individuals, organised by season, and the number of photos when each individual was identified without relying on their tags.

Fox	Territory	Season	Total *N* photos identified	*N* (%) photos where tags not visible
Holly	T1	Spring	191	30 (16%)
Poppy	T1	Spring	756	122 (16%)
Orchid	T1	Summer	1410	280 (20%)
Saffron	T6	Summer	1007	236 (23%)
Cayenne	T6	Autumn	61	11 (18%)
Ginger	T6	Autumn	337	54 (16%)
Rosemary	T6	Autumn	128	22 (17%)
Hazel	T1	Winter	766	166 (22%)
Heather	T1	Winter	287	111 (39%)
Strawberry	T7	Winter	629	110 (17%)

### The impact of fewer photos

To test the effects of fewer photos per individual and/or failing to identify all the photos, we used 125,716 photos of identified resident, and 12,613 photos of identified non-resident, foxes ≥ 5 months old. Deleting 10%, 20%, etc up to 90% of photos at random showed no effect on the number of residents we identified in any season (SP 36; SU 31; AU 43; WI 42), probably because we started with such a large number of photos of each resident fox (mean ± SE: SP 1083 ± 118; SU 1050 ± 88; AU 709 ± 55; WI 564 ± 43).

In contrast, there were increasing effects on the estimates of the number of non-residents with reducing numbers of photos (Table 3); this was probably due to the lower number of photos of each individual at the outset (mean ± SE: SP 80 ± 31; SU 92 ± 33; AU 43 ± 11; WI 47 ± 12); the total numbers of non-residents recorded each season were 52, 28, 80 and 108 respectively. The proportion of non-residents identified each season when sample sizes were reduced by 50% and 90% was: SP 88% and 60%; SU 100% and 75%; AU 90% and 54%; WI 88% and 56% i.e. there were no seasonal effects.

**Table 3:** The number of non-resident foxes ≥ 5 months old that were identified on each territory each season after deleting an increasing proportion of photos.

Territory	Season	Mean N photos per individual	Median N photos per individual	% photos remaining: number of individuals identified									
				100%	90%	80%	70%	60%	50%	40%	30%	20%	10%
**1**	SP	483	370	4	4	4	4	4	4	4	4	4	4
	SU	265	265	2	2	2	2	1	2	2	2	1	1
	AU	40	5	5	5	5	5	4	4	4	4	3	3
	WI	72	6	9	9	9	9	9	7	8	7	5	3
**2**	SP	47	2	7	7	7	7	6	4	4	4	3	3
	SU	297	297	2	2	2	2	1	2	2	1	1	1
	AU	18	4	4	4	4	3	3	4	2	3	2	2
	WI	8	5	15	15	13	14	14	14	12	11	9	10
**3**	SP	14	4	7	7	5	5	6	6	6	6	5	4
	SU	5	6	3	3	3	2	3	3	3	2	2	2
	AU	10	3	12	11	11	10	10	11	8	7	7	3
	WI	16	4	16	16	16	14	14	12	13	13	7	7
**4**	SP	41	33	5	5	4	4	5	5	4	3	5	4
	SU	0	0	0	0	0	0	0	0	0	0	0	0
	AU	25	6	10	10	10	9	8	8	8	7	8	5
	WI	28	6	13	13	13	13	12	11	13	12	8	9
**5**	SP	46	16	16	16	16	16	16	15	15	15	13	10
	SU	45	24	10	10	10	10	9	10	9	10	8	8
	AU	7	5	16	16	16	16	14	15	14	12	8	8
	WI	40	10	19	19	19	19	19	17	19	18	13	13
**6**	SP	24	9	12	12	12	12	10	12	12	10	9	6
	SU	70	20	8	8	7	8	8	8	8	7	7	7
	AU	70	18	26	26	26	25	25	25	24	24	20	17
	WI	83	30	23	23	22	22	23	23	21	20	18	15
**7**	SP	1	1	1	1	1	1	1	0	0	0	0	0
	SU	23	4	3	3	3	3	3	3	3	3	2	2
	AU	13	3	7	7	6	5	6	5	5	3	4	5
	WI	6	3	13	12	13	12	11	11	12	5	9	3
**Mean** **SE**				**9.57** **1.27**	**9.50** **1.27**	**9.25** **1.26**	**9.00** **1.25**	**8.75** **1.24**	**8.61** **1.22**	**8.39** **1.20**	**7.61** **1.17**	**6.46** **0.95**	**5.54** **0.83**

No non-residents were identified in territory 4 during the summer survey.

## Discussion

We have demonstrated that, contrary to earlier reports, individual foxes (and potentially other species with subtle markings) can be identified reliably, given a suitable study design that minimises the risks of identification errors due to subjectivity [10]. We adapted every aspect of our methodology to facilitate photo ID by maximising the quantity and quality of photos we could obtain, and used a variety of measures and individual identification sheets to enable a single observer to acquire the expertise needed to identify the individual in 99% of 170,923 timestamped camera-trap records of foxes ≥ 3 months old.

The key question in any camera-trapping study is ‘does this photo show an animal we have identified already, or is it a new individual?’ An earlier trial to test whether it was possible to identify foxes from camera-trap photos was based on a very small number of photos of random foxes from a variety of locations, with no spatiotemporal information [62]. Such a study design ignores the basic principles of camera-trapping surveys, which are conducted in defined areas for specified lengths of time, producing an archive of images for each identified individual, against which new photos can be compared. Only experienced observers can correctly identify photos of the same individual taken some time apart [28]; in the earlier study on foxes, some photos were taken in different seasons and it was suggested that the observers might consider a number of seasonally variable identification features when making their identifications [62]. They were also provided with photos from one side only, making it impossible to identify foxes photographed from different sides [80]: most of the identifying features we used were asymmetrical. So it is unsurprising that the earlier study produced a variable, but high, number of false matches [62]; false mismatches were not examined, despite their potential impact on behavioural and ecological studies.

Nor were the people used in the earlier trial of fox photo IDs trained prior to the study even though this can improve inter-observer agreement when identifying species with subtle markings [81]; they were selected for their experience of ‘working with foxes’ or ‘identifying individuals from photographs’ [62]. However, researchers with photo ID expertise of one species may not be familiar with the subtle features of another species [82], and researchers who have simply ‘worked with foxes’ may not necessarily recognise key differences in appearance. Animals look very different in real life and in photos, especially under different environmental conditions; experience of photo ID of the target species is critical for accurate identification [25].

Other studies that used similar study designs to match a limited number of photos out of context, or with limited background information and/or using observers with limited training and/or relevant expertise, have also produced high levels of inconsistency [83,84]. This is hardly surprising: even when comparing photos of humans, it can be remarkably difficult for observers to determine whether two photos of unfamiliar faces depict the same or different people, and the same observer frequently made different identification decisions on subsequent days [85]. Even though accuracy and consistency are key measures of reliability in photo ID studies, previous studies have largely focussed on inter-observer agreement, which is only a measure of accuracy when testing an observer’s ability to identify known individuals. This is seldom the case, as in the previous study on foxes [62]. The consistency of individual observers is rarely reported.

Since many previous photo ID trials are not applicable to field studies of species with more subtle markings, below we highlight some of the issues to consider when designing camera-trapping studies where individuals need to be identified.

### Techniques for identifying subtly-marked species

#### The need for a range of identifying features

Species with spot or stripe patterns can be identified reliably from a single feature, such as a distinctive neck pattern [8] or whisker spot patterns [86–89], and often from a single photo, although camera angle can still have a significant impact on the proportion of correct matches, particularly when using automatic software [89]. However, identifying individuals of species with more subtle markings requires multiple images taken from different angles, under varying environmental conditions and in different seasons, to build up a profile of unique characteristics. Too few photos of each individual will preclude detection of sufficient distinguishing features, particularly of non-residents and other animals captured infrequently [90]. More identifying features became apparent as we reviewed more photos, and observer confidence in identification increases with photographic sample size [91].

Using a combination of body features in subtly-marked species also helps safeguard against partial images, and enabled us to identify a greater proportion of photos than would have been possible when relying on a limited number of features, some of which may not be visible from every camera angle and/or environmental condition [92]. Identifying subtly-marked species is complicated when using infrared cameras [45]: colour images are generally required, and of a quality such that the image can be enlarged to confirm the presence of inconspicuous features, such as the small white mark on the upper foreleg of the fox shown in Fig 3C and 3D.

While artificial marks may be useful in aiding identification, we could not see the ear tags in a fifth of the photos of tagged animals; in some cases this was because the head was obscured, but in many the head was visible but the tags were not because of the angle of either the picture or the fox’s ears. So just relying on the tags would have risked a high rate of false mismatches; similar problems would arise with a limited number of natural features. Most importantly, though, while artificial marks speeded up the identification of some animals, it did not influence the number of foxes that we could identify.

However, radio-collaring and/or tagging some foxes in our survey area prior to installing the camera traps helped establish territory boundaries: this greatly facilitated the selection of camera-trap sites. Knowing territory boundaries also speeded up individual identification by aiding the interpretation of spatiotemporal information. While location cannot be used in isolation to identify an animal, it can provide valuable supporting information when eliminating an individual during the matching process and deciding whether there is more than one individual with similar markings. This was particularly helpful in our study where foxes within social groups, and in adjacent social groups, were often related [65] and so could be similar in appearance. The same is likely to apply to other group-living species. Studies that manage to identify subtly-marked species generally combine body features with spatiotemporal location [93–95], reinforcing the importance of not comparing photos out of context.

#### Experience is essential

Over the past 100 years, volunteers have increasingly been engaged in large‐scale monitoring projects [96], an activity now widely branded as ‘citizen science’. While volunteers are primarily used to collect data [97], citizen science has great potential to engage members of the public in the scientific process [98], and their contribution to camera-trapping studies can enhance the quantity and geographical extent of data collection for conspicuous, wide-ranging species [99]. However, while citizen science can be used to identify the species of commoner large mammals [100], even professional wildlife biologists performed poorly when asked to identify the species of small to medium-sized mammals in camera-trap photos [101].

Identifying individuals of subtly-marked species is likely to be even more challenging for citizen scientists. We used a single observer since most individual differences and identifying features only become obvious with increasing familiarity with the species, study area and study design. It takes time to develop this familiarity. If more than one person is used to identify photos, they should follow a step-wise protocol, have the same agreed identification criteria to minimise error, detailed identification sheets as described here, and training to test the accuracy of identifications of known individuals [90,102]. Inter-observer agreement is not necessarily accuracy, and true error rates cannot be determined without testing the ability of observers to identify photos of known individuals [10]. We used a number of independent measures to test the accuracy of JD’s photo IDs and, as we have already stressed, it is also important to test the consistency of individual observers.

#### Time period of surveys

For subtly-marked species, identifying features can appear very different with seasonal variations such as moult, fur wear and changes in body condition. In addition, an animal may acquire an injury or disease that changes its appearance. A camera-trapping design based on short intense survey periods with several camera traps on a territory, rather than extended surveys with one or two widely-spaced cameras, facilitates the collection of lots of photos of each individual to compile a profile and minimise the risk of false mismatches due to seasonal and other changes. Allied to this, identifying photos in chronological order allows temporal changes in individual appearance to be tracked, thereby reducing misidentifications [35,37]: photo ID is more accurate when matching photos taken close in time [58,103].

#### Understanding the importance of heterogeneity

Accuracy may be more important than identifying every capture for studies on long-distance movements and residency, and so discarding poor-quality images from the matching stage minimises the potential for incorrect assignment of individuals [99,104,105]. In such studies, peer-review systems are a useful means of quality control [106], thereby reducing the potential for human error to bias results.

Automated systems are increasingly being used to avoid the perceived subjectivity in photo ID studies, and to minimise the time spent identifying camera-trap photos. Potential computer tools include: techniques to help multiple users reach identification agreement [36]; software to pre-screen and reject most image pairs as potential matches [107]; statistical methods to account for unilateral images [80]; software to manage photo processing [108]; filters to test between target and non-target recordings [109]; and deep learning to identify, count and describe basic behaviours [33]. However, programs for the management and analysis of camera-trap data are not synergistic and each has advantages and disadvantages [102].

While automated software is beneficial in studies where discarding photos will have minimal impact on the interpretation of results, it may impose severe constraints on, and lead to errors in, population and behavioural studies. In our analyses, failing to identify non-residents and recognise that they were not part of the social group would have had a substantial effect on estimates of density, occupancy and patterns of territory use, and social group composition [79,110]. Understanding the social status and residency of an animal is also fundamental to interpreting a wide range of behaviours [70]. This is a particular problem for subtly-marked species if automatic software eliminates a significant number of photos.

In our study, we obtained far more photos of resident than non-resident foxes, although non-residents outnumbered residents, especially in autumn and winter [79]. Accurate identification of each individual, and its residency status, was key to understanding the social system of red foxes [70,79]. Using automated or manual systems that focus on clear images, a few obvious features, or other pre-selection criteria would have significantly under-estimated the number of non-residents on each territory because there were fewer photos of each animal to facilitate identification. Hence the importance of ensuring that even individuals with a single, or few, photo captures were still confident identifications. However, we acknowledge that we cannot dismiss the possibility that there were a few false mismatches in our photo IDs.

There were also large seasonal differences in the number of photos we obtained of each individual for both residents and non-residents, despite the consistency in data collection protocols across seasons; for both residents and non-residents there was roughly a two-fold difference between spring/summer and autumn/winter in the number of photos per individual. This was surprising, since the number of camera days/photographs provides a measure of comparative density for some species [111]. In our study seasonal differences in the number of photos per fox for both residents and non-residents reflected changes in behaviour, not density [79].

Few camera trapping-studies acknowledge the potential biases due to sampling protocols, seasonal changes in the behaviour of their study species, and the locations of their camera traps. For instance, although our estimates of group sizes were based on intensive 40-day surveys, these only covered ~ 44% of each season. Many of the non-residents we recorded in autumn were males that were probably making exploratory trips prior to dispersal, and in winter they were males seeking extra-group mating opportunities [79]. Both behaviours involve relatively short visits and suggest that other non-residents may have visited each territory in autumn and winter when we were not camera-trapping. So despite collecting large numbers of photos, both on each territory and of each individual, we may still have underestimated the number of non-residents (but not residents) in autumn and winter.

We positioned camera traps at the most productive food patches in each territory [70]. While this enabled us to identify all the residents on each territory, non-residents such as dispersing males keep to peripheral parts of territories and so will rarely visit food patches within territories, whereas males looking for extra-group copulations make directed movements into the core areas of territories [112]. So there will be inherent biases in our captures of non-residents. For residents, seasonal changes in the number of photos are likely to reflect, at least in part, changes in movement and foraging behaviour, and hence patterns of territorial use [79]. Unless there are enough photos to account for behavioural changes by residents, this may lead to underestimates of population size or biases in behavioural studies.

Randomly removing photos of animals to explore the effects of sample size can only be illustrative. In reality, it is highly likely that resident animals, which are seen more often, would have a higher chance of being identified, whereas non-residents would be more likely to be ‘unidentified’ because they are captured less often and so the observer would be less familiar with each individual’s features. Thus it is probable that the effects we illustrate here would have a greater impact on estimates of the numbers of non-residents. While we found no effects on our estimates of residents, this was because of the very high number of photos we had of each individual and residency status had been established with the full sample size. Most camera-trapping studies have fewer photos of each individual, often far fewer, e.g. [113,114] and, as sample sizes decline, it becomes increasingly difficult to establish whether all animals have been photo-captured, the social status of each animal, and which individuals are, and are not, residents, especially if this involves the use of a sighting threshold [79]. Many camera-trapping studies assume that all the animals photographed are residents, whereas in our study most of the animals we identified were non-residents. Having too few photographs to establish the residency of each individual, and/or failing to identify the animal in a proportion of each photographs, is likely to have a significant effect on population estimates.

#### The need to avoid influencing behaviour

The temptation is to use lures (scent and/or food) to increase photo capture rates [115,116], and thereby facilitate identification, even though this can have a significant impact on the behaviour of the study species. Regular food replenishment can also incur considerable investment of time and expense [117]. While it is rarely done in camera-trapping studies, but see [118], it is essential to quantify the impact of lures on both the focal and other species, since they can also change the behaviour of competitors [119].

We used long-established provisioning regimes in suburban gardens, thereby avoiding the risk of influencing the behaviour of our study population. The high rate of extraterritorial foraging by foxes [79] was part of the existing social system, not a response to our study. We also benefitted from working in gardens, because the environment is easy to manipulate to enhance image quality, e.g. by keeping grass mown short to increase the visibility of paw markings. Scattering food increased the time an animal spent in the locality, improved image quantity, quality and thereby identifiability [24,38], and increased the opportunities to obtain multiple photos from different perspectives [38]. This mitigated the need for multiple cameras [90].

### Conclusions

We have shown that carefully designed survey techniques permit the individual identification of subtly-marked species. No single camera deployment method will suit all situations [120], but the accuracy of individual identification of subtly-marked species is facilitated by camera-trapping techniques that yield large numbers of high resolution, colour images from multiple angles under varying environmental conditions. We emphasise the importance of choosing camera sites and settings to maximize capture rates, understanding how camera trap locations may bias results, and the impacts of seasonal changes in behaviour on camera-tapping results. While lures facilitate the collection of a number of photos of the same individual from different angles, they may bias density estimates and behavioural studies. Camera-trap images should be entered into a database alongside coordinates and timestamps so that spatiotemporal information can be used to reduce the number of potential matches between image pairs in the database.

It is unlikely that currently available automated systems could be used to identify foxes with the accuracy we needed or achieved, especially since different features were used for each animal, the features were often inconspicuous, and appearance varied with environmental conditions. Although identifying the foxes manually was extremely labour-intensive, it was critical to advancing our knowledge of fox population ecology and social behaviour. Crude estimates of fox density [121] do not facilitate effective management: social networks highlight the role of particular individuals in the spread of disease and social information [122,123], and understanding individual differences in behaviour is essential to advance population and behavioural studies [124]. Very precise information is needed to establish social group sizes in red foxes, and small changes in the information used can have a significant effect on estimates of both group size and composition [79]. Similarly, detailed information is needed to understand the interrelationships of social groups, the effects of mortality on group composition [70,79], and for how long such perturbations influence group dynamics [70,79,125–127].

Camera-trapping studies have focussed on the proportion of photos that can be identified, whereas the information lost where an individual cannot be identified in a photo is at least as important. Recent studies have examined the impact of incomplete observations on social network studies [128,129]. Any photo ID system that eliminates a significant number of photos due to image quality, or failure to identify the individual, also risks losing a great deal of important information in population studies and is likely to lead to erroneous conclusions in a range of behavioural studies.
